# *“People look and ask lots of questions”:* caregivers’ perceptions of healthcare provision and support for children born with cleft lip and palate

**DOI:** 10.1186/s12889-018-5421-x

**Published:** 2018-04-16

**Authors:** Phumzile Hlongwa, Laetitia C. Rispel

**Affiliations:** 1 School of Oral Health Sciences, Faculty of Health Sciences, University of the Witwatersrand, Johannesburg, South Africa; 2 School of Public Health, Faculty of Health Sciences, University of the Witwatersrand, Johannesburg, South Africa; 3 Centre for Health Policy & DST/NRF SARChI Chair, School of Public Health, Faculty of Health Sciences, University of the Witwatersrand, Johannesburg, South Africa

**Keywords:** Cleft lip and palate, Parents, Caregivers, Healthcare, Children, Social support, South Africa

## Abstract

**Background:**

Clefting of the lip and/or palate (CL/P) is amongst the five most common birth defects reported in South Africa. The emotional impact on parents at the birth of their new-born with CL/P could affect parent-child relationships. In light of insufficient scholarly attention parental experiences and perceptions, this study reports on caregivers’ perceptions of health service provision and support for children born with cleft lip and palate in South Africa.

**Methods:**

The study setting consisted of 11 academic hospital centres situated in six of South Africa’s nine provinces. At each of the academic centres cleft clinic, five to ten parents or caregivers were selected purposively. Participants were interviewed, using a semi-structured interview schedule that elicited socio-demographic information, explored the family experiences of having a child with CL/P, and their perceptions of care provision and support services available. The interviews were analysed using thematic content analysis.

**Results:**

Seventy-nine participants were interviewed. Their mean age was 33.3 years (range 17–68 years). The majority of the parents were black African (72%), unemployed (72%), single (67%) and with only primary school education (58%). The majority of the children were male, with a mean age of 3.8 (SD = ±4.3) years.

Five broad themes emerged from the interviews: emotional experiences following the birth of a child with cleft lip and palate; reactions from family, friends or the public; the burden of care provision; health system responsiveness; and social support services.

Caregivers reported feelings of shock, anxiety, and sadness, exacerbated by the burden of care provision, health system deficiencies, lack of public awareness and insufficient social support services.

**Conclusions:**

The findings have implications for the integrated management of children with cleft lip and/or palate, including information to parents, the education and training of healthcare providers, raising public awareness of birth defects, and social support.

**Electronic supplementary material:**

The online version of this article (10.1186/s12889-018-5421-x) contains supplementary material, which is available to authorized users.

## Background

Congenital anomalies are a major public health concern worldwide, because of their contribution to infant and childhood mortality, chronic illness and disability [1]. Clefting of the lip and/or palate (CL/P) is a major structural congenital defect that requires surgical intervention and has significant medical, social and psychological consequences for affected individuals and their families [1]. The worldwide prevalence is estimated at 1 in 700 live births, but this varies according to geographic location, socio-economic status and ethnic background [2–4]. In South Africa, cleft of the lip and/or palate (CL/P) is amongst the five most common birth defects reported in South Africa [5], with the incidence of CL/P ranging from 0.1 to 0.4 per 1000 live births [5–7].

Research on CL/P is extensive, and includes studies on prevalence, determinants and associated anomalies [5, 6, 8–12], the diagnosis and medical management of CL/P [13–18]; feeding problems as well as speech, hearing, and dental complications of individuals with CL/P [19–23]; long-term health outcomes [19–24]; and the expressed communication needs and psychosocial issues faced by individuals with CL/P and their parents [25–40].

Most of the research on experiences and perceptions of parents of children with CL/P is in high-income countries (HIC) [15, 29, 30, 33, 34, 37]. In these countries, studies have found that parents experienced varying degrees of shock, anger, denial, distress and anxiety, and a sense of “loss of control” over a CL/P birth [15, 29, 30, 33, 34, 37]. Mothers expressed feelings of guilt because they carried the infants to term [15]. In these studies, parents underscored the importance of appropriate and accurate information about CL/P and health and social support with their children’s condition at birth [26, 28, 30, 37, 40]. Some studies found that parents who had a delay in obtaining information from health professionals searched for this on the internet, which exacerbated their anxieties and distress [15].

In many low- and middle-income countries (LMIC), infectious diseases dominate the causes of infant and child mortality, and congenital anomalies account for a relatively small proportion of under-five mortality [38, 41]. Hence, there is a dearth of research on congenital anomalies in LMIC. Nonetheless, there is an emerging body of literature that describes parental emotions of shock, distress and anxiety [28, 37, 42, 43]. A study in Brazil found that frequent hospitalizations of CL/P children add to the parental feelings of stress, fear, anxiety and helplessness with the stress levels of parents higher prior to their children’s corrective surgery [43]. Mothers of children with clefts expressed hurt after birth and attributed the cause of oro-facial clefts to evil or ancestral spirits or the will of God. The parents also talked about the stigma experienced, as their children with oral clefts were often treated as outcasts in their communities [35, 42].

In a 2002 study from Pretoria, South Africa, in which the mothers’ experiences of pre- and postnatal diagnosis of clefts were explored, it was discovered that the timing of diagnosis did not influence the need for emotional support, information, interaction with other parents of children with clefts, and a multidisciplinary team approach to care and treatment [37]. A 2014 study in a private hospital on parents’ experiences of children with CL/P found that parents were satisfied with treatment they received, but expressed dissatisfaction with financial support for treatment, transport to the facility, and lack of parental education and information [28]. However, the experiences and views of parents in the public health sector might be different.

There remains a paucity of research on CL/P in South Africa, and the perceptions and experiences of parents of children with CL/P, especially studies that are representative of all provinces, of urban and rural settings, and of the public health sector. In light of insufficient scholarly attention on the psycho-social aspects of CL/P as a congenital anomaly this study focused on parental experiences, of a child with CL/P, and parents’ perceptions of health care provision and support for their children in the public health sector. It is part of a large doctoral study on the epidemiology and care provision of CL/P individuals in South Africa.

## Methods

The Human Research Ethics Committee (HREC) (Medical) of the University of the Witwatersrand in Johannesburg provided ethical approval to conduct this research (M1501536). Permission was also obtained from the relevant health care authorities. We adhered to standard ethical procedures, which included study information sheets, voluntary participation, informed consent, confidentiality, and anonymity in the management of data and reporting of study findings.

We restricted the study to the public health sector in South Africa, as it provides health care services to the majority of the population in the country. The study setting consisted of all the 11 academic hospital centres that provide specialised care to individuals with CL/P situated in six of South Africa’s nine provinces. These centres were selected because they are referral centres that provide specialised treatment to children born with CL/P and multi-disciplinary teams of health professionals are available at these centres.

Although the number of participants in qualitative studies depends on the point at which data saturation is reached [44], we considered it important to obtain the views of parents at each of the specialised treatment centres. At each of the CL/P care centres, we aimed to select a minimum of five and maximum of ten parents or caregivers of children with CL/P, to take account of size and the catchment area of the specialised treatment centre. We selected caregivers purposively among those who attended the CL/P clinic on the fieldwork day, and selected them as they arrived at each centre.

On the fieldwork day, the principal investigator (PI) approached caregivers present at the relevant specialised treatment centre, explained the study verbally, handed an information sheet to each participant, and requested participation in the study. Those individuals who agreed to participate in the study were enrolled and in those instances where both parents were present at the clinic appointment, the mother was interviewed.

Following informed, written consent, the PI used a semi-structured questionnaire (Additional file 1), designed in English, to conduct the interviews with the caregivers. We designed the questionnaire specifically for the study. The questionnaire contained 27 questions divided into three parts: socio-demographic information, the parental experiences of having a child with CL/P, reactions from families, friends and the general public; perceptions of health care provision; and the availability of support services.

At each centre, the PI conducted the interviews in a private room adjacent to the consulting room. The interviews were conducted in English, with clarification of terms into one of South Africa’s local languages, where relevant, as the PI is fluent in local languages. Each interview lasted around 30 min. The responses of participants were written down verbatim, typed up and saved as individual Microsoft Word © documents soon after the interview.

The interviews were analysed using thematic content analysis [45]. The first step in the analysis was to look at participants’ own words and phrases and without preconceived notions or classification. We then examined the language used by each participant in response to the following questions: What do the responses tell us about the experiences, feelings and perspectives of parents or caregivers? What is emerging about the experiences associated with having a child with CL/P, health care provision or social support services?

To ensure trustworthiness, two additional researchers (an experienced qualitative researcher with health system experience and the primary supervisor) participated in the development of the codes by reading the parent responses independently from the PI in order to establish inter-coder agreement [45, 46]. Once the initial analysis was completed, the team liaised to discuss the codes generated independently, and to reach agreement on the codes and themes. Following inter-coder agreement and the themes, the PR analysed the interviews [45].

## Results

### Socio-demographic characteristics of participants

All parents or caregivers that were recruited at the specialised treatment centres, agreed to participate in the study; hence a 100% response rate was obtained. The study recruited 79 participants: 68 were biological parents, four were guardians, five were relatives, and there was one foster parent and one caregiver with a child from an orphanage. The mean age of the participants was 33.3 years (range 17–68 years). The majority of the participants were black African (72%), unemployed (67%), single (67%) and with only primary school education (58%). The majority of children were male, with a mean age of 3.8 (SD = ±4.3) years (Table 1).

**Table 1:** Social and demographic characteristics of caregivers

Characteristics	*N* = 79
Mean age in years (Standard Deviation)	33.3 (17.2)
Relationship to CL/P Child	
Biological Parent	68 (86.1%)
Foster Parent	1 (1.3%)
Relative	5 (6.3%
Guardian	4 (5.1%)
Caregiver	1 (1.3%)
Race	
Black African	57 (72.2%)
Coloured	11 (13.9%)
Indian	2 (2.5%)
White	9 (11.4%)
Employment status	
Employed	22 (27.8%)
Unemployed	57 (72.2%)
Education status	
Primary school	46 (58.2%)
Secondary school	31 (39.2%)
Tertiary	2 (2.6%)
Marital status	
Married	26 (33%)
Single	53 (67%)
Children in each family including CL/P Child	
Number of children – mean (Standard Deviation)	2.2 (±1.3)
Mean age in years (Standard Deviation)	3.8 (±4.3)

### Caregivers’ experiences and perceptions of health care provision and support services

Although overlapping, five broad themes emerged from the responses of study participants to the open-ended questions: emotional experiences following the birth of a child with cleft lip and palate; reactions from family, friends or the public; the burden of care experienced by caregivers; health system responsiveness and social support services (Table 2).

**Table 2:** Caregivers’ experiences and perceptions themes

Theme	Description
Emotional experiences of having a child with CL/P	Mother • Self-blame • Shock • Feeling Scared • Crying • Acceptance of the child Partner • Acceptance of the child • Denial of the child • Supportive Concerns about • Speech • Schooling • Long term well-being
Reactions from family, friends and the public	Family and friends • supportive Public • Gossiping • Staring • Asking lots of Questions
Burden of care	• Feeding needs and challenges • Need for full-time and extra care • Many hospital visits - Transport costly - Frequent time off from work - Inability to work
Health system responsiveness	Health provider attitudes and behaviour • Pleasant attitude • Rude behaviour • Caring attitude Treatment provision • No payment for treatment • Satisfied with treatment • Lack of resources for mothers Inadequate or lack of communication • Little or no Information • Inadequate Counselling • Little or no explanation
Social support services	• Lack of public awareness • Nuances of counselling

### Emotional experiences following the birth of a child with cleft lip and palate

A major theme that emerged from the caregivers’ responses was the emotional experiences following the birth of a child with cleft lip and palate. The birth of a child with CL/P evoked mixed feelings of shock, anxiety, distress, worry, sadness, and misery among parents because they did not know that their new-born baby would be born with a cleft lip and palate. Many mothers reported that they cried uncontrollably. At the same time, they also experienced happiness about their live born children, and some expressed a combination of feelings of shock and acceptance of the child “as a gift from God”.

I was shocked because I have not seen a cleft before, but I accepted the baby as a gift from God (Parent 6; Site 8, KwaZulu Natal Province).

Another parent reported screaming and crying when she was shown her baby at birth:

I screamed and was shocked when they showed me the baby. I asked: “what happened to my baby?”… I cried. (Parent 10; Site10, Free State Province).

Most parents blamed themselves and expressed feelings of guilt because of the birth of a baby with a cleft lip and palate condition, especially those parents who reported smoking and alcohol during pregnancy.

I blame myself because I was smoking. I caused my baby to have the cleft (Parent 6, Site 6, Western Cape Province)

Some parents ascribed the CL/P to delay in the first antenatal care visit to a health care facility or because they desired a baby with a different sex, than the baby who was born.

I am guilty because I went to hospital later and not early enough when I was pregnant (Parent 5; Site 5, Gauteng Province).

I think it is my fault because I wanted a girl so God did the opening in the mouth because he is a boy (Parent 1; Site 11, Limpopo Province).

The interviews revealed that there were differences in the reactions between mothers and fathers. In some instances, the fathers were reported to be supportive and loving.

The father loves her even though it was the first time he sees this since no one in our family has it (Parent 2; Site 6, Western Cape Province).

The father is supportive even though he does not live with us in the same house but he gives us money for milk, clothes and hospital visits (Parent 9; Site 4, Gauteng Province).

However, there were instances where mothers reported that the fathers rejected them and the babies. Some fathers denied the babies as theirs, either claiming that they do not “give birth to disabled children”, or left and never returned.

The father denied the child saying he does not want an abnormal child (Parent 4; Site 8, KwaZulu Natal Province)

His father denied that it is his child immediately when he saw that he has a cleft condition (Parent 1; Site 9, Eastern Cape Province).

Many parents also expressed concerns about speech difficulties experienced by children with CL/P, which in turn impacted on future schooling and peer acceptance. They wished that their children should attend normal school and not a special school for children with disability. They also expressed concerns about the overall well-being of the child, hoping that the medical team would help their babies to become “normal”:

We wish that the hole must be closed inside [his mouth] so that he is able to speak properly as he will be 4-years old next year and he must attend preschool (Parent 3; Site 11, Limpopo Province).

I wish that my child must be well; be able to go to school and be successful (Parent1; Site 9, Eastern Cape Province).

I wish that he must have normal schooling; today the medical world is much better equipped to handle this condition and I am confident that he will be helped (Parent 4; Site 4, Gauteng Province).

I do not know what to say to him when he is old, my son is scarred for life; I wish that he must be helped to be normal (Parent 4; Site 7, Western Cape Province).

### Reactions from family, friends or the public

The nature and extent of support from family and friends was the second theme that emerged from the interviews. In some instances, caregivers expressed appreciation to their families and friends for their caring and understanding.

My family accepted my baby and they were supportive (Parent 5; Site 11, Limpopo Province).

There is no conflict in our family because of the child, the family has been working together to make sure that everything goes well with him (Parent 6; Site 8, KwaZulu Natal Province).

In other instances, mothers reported alienation from people they considered as their friends, who distanced themselves. This was interpreted by the mothers as their friends not wanting to hurt them [the parents] because they had not seen the cleft before.

In the case of children with isolated cleft palate, the parents indicated that they were not ashamed of their children in public because the hole was inside the mouth and could not be seen by other people. However, the majority of parents said that they were not comfortable or happy to show their children in public, because of stares, hurtful comments and/or gossiping. They expressed concerns about post-natal clinic visits indicating that mothers with “normal” children were always looking at their cleft children with curiosity.

I was very much ashamed especially in the clinic where other mothers were always looking at her; it was not nice (Parent 1; Site10, Free State Province)

Many parents reported that they only felt comfortable with the child in public after the lip surgery was done, commonly after 3 months of birth.

I was not comfortable with him in public before surgery because people look and ask lots of questions, but now [after surgery] it is okay to show him in public (Parent 1, Site 7, Western Cape Province).

I was not [comfortable], until the surgery was done; I keep her in the house as the whole village was gossiping (Parent 2; Site 8, KwaZulu Natal Province)

We did not show him in public until he was operated. We were scared of germs and also of people looking (Parent 5; Site 11, Limpopo Province)

I have accepted to show him in public places now that he is 8-years old but when he small it was very embarrassing because people were asking what is this (Parent 7; Site 4, Gauteng Province)

### Burden of care provision

Another major theme that emerged from the interviews with parents was the burden of caring for a child with cleft lip and palate. This burden was expressed through the comments on the feeding needs and challenges of their children; the need for extra care or attention, and the necessity of “many” hospital visits to different health care providers.

Feeding difficulties were a constant thread in most of the interviews. The mothers reported that they were scared of the child choking during feeding because of the hole between the mouth and the nose. The comments below illustrate the caregivers’ distress about feeding of their children:

I compared this baby to the older child who was normal- feeding is a problem as milk comes through the nose (Parent 2; Site 6, Western Cape Province).

Feeding is a problem because milk is coming out through the nose so we have to watch her all the time so that she is not choking (Parent 1, Site 10, Free State Province)

Caregivers reported that children with cleft lip and palate need greater attention because of feeding difficulties, the perceived vulnerability of these children relative to “normal” children, and frequent visits to health care facilities. Parents reported that they were not comfortable with leaving their children in the care of others. One said:

I have to be with my child all the time and I do not trust other people with her (Parent 6; Site10, Free State Province).

Others commented as follows:

This child needs more attention as he is a special child so I dedicate all my time for him to be well (Parent 4, Site 11, Limpopo Province)

Yes, my child needs more attention from me as my family were scared of her so I was the only one feeding her (Parent 2, Site 4, Gauteng Province)

Frequent hospital visits added to the burden of caring, because some parents in more rural areas had to travel long distances to specialised treatment facilities. Furthermore, the majority of parents used public transport which was costly especially to those who were unemployed. Others raised concerns that the frequent hospital visits and requesting time off from work affected their employment.

I had to take two weeks leave for hospitalization when my child was operated. Taking time off from work for the hospital appointment affected my way of life, I don’t get paid for being absent so many times from work (Parent 3; Site 5, Gauteng Province).

Some parents reported that they were unable to work or look for work, because they had to take care of their children.

### Health system responsiveness

Some mothers reported that the treatment they received was good and that both nurses and the doctors provided information, counselled them, and reassured them, which made it easier to accept their babies with CL/P.

I received good information from the nurses and doctor, they reassured me that my baby will be okay (Parent 10; Site 4, Gauteng Province).

The hospital doctors gave us the information about cleft, they told us that many other children are born like this and he [the baby] will be okay (Parent 1; Site 7, Western Cape Province).

The nurses and doctors were very nice to me and my child and they explained that my child is not the only one with this condition, so he will be helped (Parent 7; Site 8, KwaZulu Natal Province).

The comments about good quality of care tended to be at academic hospitals in large cities, because of the availability of a multi-disciplinary team of health professionals to support the parents.

However, the majority of parents reported several health system deficiencies that affected the care of their babies. These deficiencies included lack of information regarding the condition of their babies at birth, lack of explanation about CL/P, and unhelpful and rude behaviour and attitudes of nurses at maternity clinics. These deficiencies contributed to their feelings of shock and distress.

I was never told until the next morning. I discovered it [cleft] myself when the baby was in the incubator. (Parent 7; Site 2, Gauteng Province).

[The cleft lip and palate] information was not enough, I used the internet and got more information about it [CL/P] (Parent 2; Site 11, Limpopo Province).

I was not treated well. They [the nurses] were talking to each other and did not tell me anything about my baby, later they informed me that my child is not complete (Parent 8; Site1, Gauteng Province).

I was treated badly by the nurses, I was placed in a separate room and I was not told about my baby while the nursing staff was gossiping (Parent 4; Site 8, KwaZulu Natal Province).

Parents reported that they were happy with the free surgical treatment provided.

The doctors were helpful to both my girls, they [the girls] have been operated for free and they look pretty (Parent 4; Site 6, Western Cape Province)

The doctors are treating all these children very well; my child’s lip was operated and I am very happy (Parent 6; Site 4, Gauteng Province)

I am very grateful to the doctors for fixing my son (Parent 6; Site 8, KwaZulu Natal Province)

I am happy with treatment even though it was delayed. He should have been operated when he was 6 months old, now he is 9 months. I was left behind by the transport to travel for treatment as it is not done in this hospital (Parent 1; Site 9, Eastern Cape Province)

### Social support services

Social support service was the fifth theme that emerged from the parents, and includes the lack of public awareness and lack of counselling received by the mothers at the birth of their CL/P child. The majority of parents reported that they have never seen a cleft before and it was the first time in their family for a child to have this condition. Parents reported lack of counselling regarding this condition at the birth of their children.

I want the doctors and nurses to make public awareness especially in rural areas. I suffered for two months and my child could have died from the milk. The nurses in the clinics should know about cleft, and that it can be treated (Parent 2; Site 9, Eastern Cape Province).

I did not know at birth that my child has a cleft. I only saw the milk coming out the baby’s nose and I was thinking that he has a small stomach and I am feeding him too much milk (Parent 3; Site 10, Free State Province).

I was not told what a cleft is and what causes it until today as I am speaking with you. You are the first person to explain cleft palate to me (Parent 1; Site 8, KwaZulu Natal Province).

Some parents reported that the hospital did not provide accommodation and lodging for them when their children were admitted for the surgical procedure. This was cited as a challenge because the parents’ homes were far from the hospital and they had to sleep on the chairs for a number of days without food and facilities for their personal hygiene.

The nurses must have patience with us. When my child was admitted for the lip surgery, I slept on a chair for seven days waiting for my child to be discharged from hospital and did not have food. I wish that the hospital must give us [mothers] a place to sleep when we wait for our babies’ surgery (Parent 5; Site 8, KwaZulu Natal Province).

## Discussion

This is one of the first, comprehensive studies that explored the socio-demographic characteristics of the caregivers of CL/P children, the family impact of having a child with CL/P, and their perceptions of health care provision and support services available at the 11 specialised academic centres in the South African public sector. The majority of study participants were single, unemployed, women who had completed primary schooling. Although we did not ask about the nature and availability of family support, the findings suggest that the responsibilities for care provision of these CL/P children fall disproportionately on these women. Studies in the United States and the United Kingdom reported that the long term implications of parenting children with clefts include higher stress levels, anxiety and depression, a risk of maternal detachment, and lower cognitive functioning of the children [15, 47, 48]. Hence, the single mothers may require additional health and social service support. Furthermore, their socio-demographic characteristics need to be taken into account in health information, education and communication programmes in the South African public health sector. The specialist multi-disciplinary cleft team and the community healthcare services can play an important role in implementation of such programmes.

This PhD research provides unique parental perspectives of the effects on the family of children born with clefts. In our study, the participants expressed varying degrees of emotional reactions similar to studies done in other countries [15, 29, 30, 33, 34, 37, 49, 50]. Amidst these negative emotions, some parents expressed the view that the child is “a gift from God”. This view may serve both as explanation of causation by a supernatural force, and as a coping strategy. A study from Nigeria also reported that Muslim parents who belonged to a specific ethnic group attributed CL/P to the “will of God”. The authors pointed out that the meaning of birth defects and the future faced by individuals with CL/P are influenced by a complex set of social and cultural factors, that should inform the provision of health care services [51].

In our study, mothers’ self-blame and feelings of guilt were both reinforced and exacerbated by the rejection of the babies’ fathers and the courtesy stigma experienced from friends, the public, and uninformed nurses in health care facilities located outside the specialised academic centres. The notion of courtesy stigma, first described by Goffman [52], is the public disapproval evoked because of the association with a stigmatised individual or group, in this case a CL/P child. In this study such courtesy stigma was expressed through staring, gossiping, and excessive questioning that made mothers feel uncomfortable and/or ashamed. A Ugandan study also found stigma narratives from mothers who reported that children with CL/P were treated as “outcasts”, while doctors reported that these children were not accepted by their communities [53]. Similarly, families may see a child with CL/P as a “family shame”, a feeling that may influence the social position of such families. A ten-year review done by the Smile Train of their work in 33 African countries found that stigma influenced case reporting, family relations, utilisation of health care services, and ability to conduct research [54]. Our findings point to the need for public awareness campaigns on CL/P, as part of the South African Department of Health’s overall strategic plan on non-communicable diseases. Nurses, who work in health facilities outside academic facilities, and in rural areas where there are no specialised treatment centres, require continuing professional education on CL/P.

The mothers gave moving accounts of the burden of caring for a child with cleft lip and palate, especially feeding difficulties before the surgery was done and the necessity of numerous hospital visits to different health care providers. Feeding problems of babies with CL/P at birth are universal, and detailed guidelines exist on feeding approaches, advice and information for mothers, and the management of complications. These guidelines could be adapted both for parents as well as for health professionals outside specialised health facilities.

The mothers’ narratives on the burden of care should be viewed within the context of the emotional impact of CL/P children, and the experiences of courtesy stigma. A study in Brazil found that frequent hospital visits added to family stress and anxiety [43]. In this study, the mothers’ narratives of the health system deficiencies were more acute in rural and non-specialised health care facilities, and this may worsen their stress. The inequities and complexities of care provision in the South African public health sector have been well described, and are illustrated through the comments on the “free and good” treatment they received, as well as the information, counselling and reassurance from doctors and nurses in the specialised academic centres. This means that there is a solid foundation to build on and opportunities to expand good practice models on information, counselling and social support to rural and non-specialised health care facilities. This is important because evidence suggests that lack of parental social support is one of the factors that influence the nature and quality of the attachment children form with their main caregivers [15, 39].

In light of the 2015 sustainable development goals that emphasise universal health coverage and inclusive societies, this study provides important information on the issues that need to be taken into account in the provision of health services that are responsive to the needs of caregivers and children born with CL/P. The study did not explore the use of traditional healers, or existing family support mechanisms. These are study limitations. Nonetheless, it is one of the first studies that explored the experiences of families with the birth of a child with CL/P, and caregiver perspectives on health care and social service provision in the public health sector. The study participants were selected purposively, and represent all the specialised academic centres in South Africa’s public health sector, rather than the views of parents at one centre. The study has generated new knowledge on health system deficiencies for individuals with orofacial clefts in South Africa, which can be used to shape future treatment efforts, both by multi-disciplinary teams at specialised centres and community-based services.

The experience of well-established CL/P treatment facilities in HICs provide a useful framework for care provision, that is applicable to resource-constrained settings [15]. These include: customised information and communication programmes that enhance the understanding of CL/P among parents; teaching parents the skills to handle and feed the baby to enhance their confidence and to reduce anxiety; forums or opportunities for parents to express their feelings and come to terms with taking care of a baby with CL/P; informing parents about treatment, care and rehabilitation of their children, and the members of the health care team; social support, which could include the child support grant, access to other parents, and parent support groups [15].

## Conclusions

This study has highlighted the perceptions and the experiences of caregivers regarding health care service provision and support for their CL/P children in the South African public health sector. The study underscores the importance of sufficient information regarding cleft lip and palate condition, supportive families and friends, and optimal functioning of the health system.

The study findings have implications for the integrated management of individuals with cleft lip and/or palate. Such management should include: addressing the information needs of parents and caregivers; the education and training of health care providers outside specialised centres; raising public awareness of birth defects, including CL/P and social support programmes.

## Additional file

Additional file 1: Caregivers’ Qestionnaire. (DOCX 40 kb)

## Abbreviations

CL/P: Cleft lip and/or palate
CLP: Cleft lip and palate
DST: Department of Science and Technology
HIC: High-income countries
HREC: Human Research Ethics Committee
LCR: Laetitia Charmaine Rispel
LMIC: Low-and middle-income countries
NRF: National Research Foundation
PH: Phumzile Hlongwa
PhD: Doctor of Philosophy
PI: Principal investigator
SARChI: South African Research Chairs Initiative
SD: Standard deviation
WHO: World Health Organization
