# Supplementary material for: “People look and ask lots of questions”: caregivers’ perceptions of healthcare provision and support for children born with cleft lip and palate
Source: BMC Public Health. 2018 Apr 16;18:506. doi: 10.1186/s12889-018-5421-x (PMC5902984; doi:10.1186/s12889-018-5421-x)
Supplement: Supplementary file 1 — Caregivers’ Qestionnaire. (DOCX 40 kb) [file 12889_2018_5421_MOESM1_ESM.docx]

**Caregivers’ perceptions of healthcare provision and support for children born with cleft lip and palate**

**Additional file 1: CAREGIVERS’ QUESTIONNAIRE**

**For official use only**

| 1. 3 | Participant number |  | \|  \|  \|  \|  \| \| --- \| --- \| --- \| --- \| |
| --- | --- | --- | --- | --- | --- | --- | --- |
|  | Study site   - CHBH…..1 - CMJAH…..2 - SBAH…….3 - DGMH……4 - PTA …….5 - TBH…….6 - RCWMCH……7 - IALCH……..8 - NMH………9 - UVH……..10 - PMHC…….11 |  | \|  \|  \|  \|  \|  \|  \| \| --- \| --- \| --- \| --- \| --- \| --- \| |
|  | Date of interview | DD/MM/YY | \|  \|  \|  \|  \|  \|  \| \| --- \| --- \| --- \| --- \| --- \| --- \| |

**STATEMENT OF CONSENT**

I have been given an information sheet and I understand the objectives of the study. I further understand that my responses will be kept confidential and that it is up me whether or not to participate in an interview. It has been explained to me that even if I choose not to participate, I should indicate **No** in the space below. My refusal to participate will in no way prejudice me**.**

I agree voluntarily to participate in the interview **(please tick).** 🞎 **Yes**

🞎 **No**

**IF YOU AGREE TO PARTICIPATE, PLEASE ANSWER ALL OF THE FOLLOWING QUESTIONS. TICK/ MARK OR CIRCLE THE BOX NEXT TO THE APPROPRIATE ANSWER.**

SECTION 1 – BACKGROUND INFORMATION

| OFFICE USE ONLY |  |
| --- | --- |
| □ | 1. What is your relationship with the CL/P child?   □Biological parent (1)  □Foster parent (2)  □Relative (3)  □Guardian (4)  □Care-giver (5)  □Other (9)specify…………………………………………………………… |
| □ | 1. What is your age in years? …………………………………………………. |
| □ | 1. Which of the parent are you?   □ Mother(1)  □ Father (2)  □ Other (9) specify |
| □ | 1. Race   Black……..(1)  Coloured…(2)  Indian……(3)  White…..(4)  Other …..(9) |
| □ | 1. What is your employment status?   □ Employed.(1)  □ Unemployed (2)  □ Other (9) specify………………………………………………………… |
| □ | 1. What sex is your CL/P child?   □Male (0)  □Female (1) |
| □ | 1. How old is your CL/P child?.................Years.......................Months |
| □ | 1. What type of cleft does your child have?……………………………………………. |
| □ | 1. How many children do you have including this one?....................... |
| □ | 1. What is your marital status?   □Single (1)  □Married (2)  □Other (9) |
| □ | 1. What is your highest education status?   □None (0)  □Matric (1)  □Tertiary education  □Other (9) specify………………………………………………………….. |

**SECTION 2 – FAMILY IMPACT**

| OFFICE USE ONLY |  |
| --- | --- |
| □ | 1. How has your work or the other parent been affected by your CL/P child?   ------------------------------------------------------------------------------------------------------------------------------------------------------------------------------------------------------------------------------------------------------------------------------------------------------------------------------------------------------------------ |
| □ | 1. Has your child required more attention from you or the other parent?   ---------------------------------------------------------------------------------------------------------------------------------------------------------------------------------------------------------------------------------------------------------------------------------------------------------------------------------------------------------------------------------------------------------------------------------------------------------------- |
| □ | 1. How have the other family members responded on your CL/P child?   ---------------------------------------------------------------------------------------------------------------------------------------------------------------------------------------------------------------------------------------------------------------------------------------------------------------------------------------------------------------------------------------------------------------------------------------------------------------- |
| □ | 1. Have you or the other parent been upset because of the CL/P child?   ---------------------------------------------------------------------------------------------------------------------------------------------------------------------------------------------------------------------------------------------------------------------------------------------------------------------------------------------------------------------------------------------------------------------------------------------------------------- |
| □ | 1. Do you or the other parent have any feelings of guilt with the birth of this child?   ----------------------------------------------------------------------------------------------------------------------------------------------------------------------------------------------------------------------------------------------------------------------------------------------------------------------------------------------------------------------------------------------------------------------------------------------------------------- |
| □ | 1. What are your most concerned issues on the child’s future?   ---------------------------------------------------------------------------------------------------------------------------------------------------------------------------------------------------------------------------------------------------------------------------------------------------------------------------------------------------------------------------------------------------------------------------------------------------------------- |
| □ | 1. Are you comfortable with your child in public places?   ----------------------------------------------------------------------------------------------------------------------------------------------------------------------------------------------------------------------------------------------------------------------------------------------------------------------------------------------------------------------------------------------------------------------------------------------------------------- |

| □ | 1. How does your partner or other children coping with this child?   ----------------------------------------------------------------------------------------------------------------------------------------------------------------------------------------------------------------------------------------------------------------------------------------------------------------------------------------------------------------- |
| --- | --- |
| □ | 1. Has your child’s condition caused disagreement or conflict in the family?   ----------------------------------------------------------------------------------------------------------------------------------------------------------------------------------------------------------------------------------------------------------------------------------------------------------------------------------------------------------------------------------------------------------------------------------------------------------------- |
| □ | 1. Do you blame yourself or the other parent for your child condition?   ------------------------------------------------------------------------------------------------------------------------------------------------------------------------------------------------------------------------------------------------------------------------------------------- |
| □ | 1. Has your child’s condition caused financial difficulties for your family?   ----------------------------------------------------------------------------------------------------------------------------------------------------------------------------------------------------------------------------------------------------------------------------------------------------------------------------------------------------------------------------------------------------------------------------------------------------------------- |

**SECTION 3 – SUPPORT SERVICES**

| OFFICE USE ONLY |  |
| --- | --- |
| □ | 1. Were you informed before birth about your CL/P child condition?   --------------------------------------------------------------------------------------------------------------------------------------------------------------------------------------------------------------------------------------------------------------------------------------------------------------------------------------------------------------------------------------------------------------------------------------------------------------- |
| □ | 1. Did you receive counseling regarding your child CL/P condition and how was it done?   ---------------------------------------------------------------------------------------------------------------------------------------------------------------------------------------------------------------------------------------------------------------------------------------------------------------------------------------------------------------------------------------------------------------------------------- |
| □ | 1. Were the causes of CL/P condition explained to you and did you understand?   -------------------------------------------------------------------------------------------------------------------------------------------------------------------------------------------------------------------------------------------------------------------------------------------------------------------------------------------------------------------------------------------------------------------------------------------------------------- |
| □ | 1. Was termination of pregnancy an option for you due to the condition of your child? or Would you have considered to terminate pregnancy should you have known during pregnancy?   --------------------------------------------------------------------------------------------------------------------------------------------------------------------------------------------------------------------------------------------------------------------------------------------------------------------------------------------------------------------------------------------------------------------------------------------------------------- |
| □ | 1. How was your CL/P child treated at birth?   --------------------------------------------------------------------------------------------------------------------------------------------------------------------------------------------------------------------------------------------------------------------------------------------------------------------------------------------------------------------------------------------------------------------------------------------------------------- |
| □ | 1. How can you describe the information you received at the birth of your CL/P child?   -------------------------------------------------------------------------------------------------------------------------------------------------------------------------------------------------------------------------------------------------------------------------------------------------------------------------------------------------------------- |
| □ | 1. What information did you receive regarding your CL/P child treatment?   ----------------------------------------------------------------------------------------------------------------------------------------------------------------------------------------------------------------------------------------------------------------------------------------------------------------------------------------------------------------------------------------------------------------------------------------------------------------------------------------------------------------------------------------------------------------------------------------- |
| □ | 1. Do you feel that having a CL/P child affected your way of life?   -------------------------------------------------------------------------------------------------------------------------------------------------------------------------------------------------------------------------------------------------------------------------------------------------------------------------------------------------------------------------------------------- |
| □ | 1. How has your CL/P child affected your relationship with your friends?   -------------------------------------------------------------------------------------------------------------------------------------------------------------------------------------------------------------------------------------------------------------------------------------------------------------------------------------------------------------------------------------------- |
| □ | 1. How has your CL/P child affected your family relationship?   -------------------------------------------------------------------------------------------------------------------------------------------------------------------------------------------------------------------------------------------------------------------------------------------------------------------------------------------------------------------------------------------- |
| □ | 1. How were your affected by CL/P child’s condition at birth?   --------------------------------------------------------------------------------------------------------------------------------------------------------------------------------------------------------------------------------------------------------------------------------------------------------------------------------------------------------------- |
| □ | 1. Were you satisfied with the information given at the birth of your child?   ------------------------------------------------------------------------------------------------------------------------------------------------------------------------------------------------------------------------------------------------------------------------------------------------------------------------------------------------------------- |

| □ | 1. Do you think your child has received necessary treatment from birth till now?   ------------------------------------------------------------------------------------------------------------------------------------------------------------------------------------------------------------------------------------------------------------------------------------------------------------------------------------------------------------------------------------------------------------------------------------------------------------- |
| --- | --- |
| □ | 1. Were you satisfied with the support given by the medical team?   --------------------------------------------------------------------------------------------------------------------------------------------------------------------------------------------------------------------------------------------------------------------------------------------------------------------------------------------------------------------------------------------------------------------------------------------------------------- |
| □ | 1. Was the treatment for your CL/P child provided at an easily accessible hospital?   ----------------------------------------------------------------------------------------------------------------------------------------------------------------------------------------------------------------------------------------------------------------------------------------------------------------------------------------------------------------------------------------------------------------------------------------------------------------------------------------------------------------------------------------------------------- |
| □ | 1. Which of the following medical team members played a role in your child’s treatment?  \| 1. Geneticist/Genetic Counsellor \| 🞎 No...0  🞎 Yes...1 \| \| --- \| --- \| \| 1. Plastic Surgeon \| 🞎 No...0  🞎 Yes...1 \| \| 1. Orthodontist \| 🞎 No...0  🞎 Yes...1 \| \| 1. ENT Surgeon \| 🞎 No...0  🞎 Yes...1 \| \| 1. Maxillo-Facial Surgeon \| 🞎 No...0  🞎 Yes...1 \| \| 1. Paediatric Dentist \| 🞎 No...0  🞎 Yes...1 \| \| 1. Paediatric surgeon \| 🞎 No...0  🞎 Yes...1 \| \| 1. Speech Therapist \| 🞎 No...0  🞎 Yes...1 \| \| 1. Professional nurse \| 🞎 No...0  🞎 Yes...1 \| \| 1. Other, please specify…………………………………………………………………………………………………………………………….. \| 🞎 No...0  🞎 Yes...1 \| |
| □ | 1. Do you have any recommendations on the care of CL/P children?   _______________________________________________________________________________________________________________________________________________________________________________________________________________________________________________________________________________________________________________________________________________________________ |

**Thank you for participating**
